# Blocking Migration of Polymorphonuclear Myeloid-Derived Suppressor Cells Inhibits Mouse Melanoma Progression

**DOI:** 10.3390/cancers13040726

**Published:** 2021-02-10

**Authors:** Christopher Groth, Ludovica Arpinati, Merav E. Shaul, Nina Winkler, Klara Diester, Nicolas Gengenbacher, Rebekka Weber, Ihor Arkhypov, Samantha Lasser, Vera Petrova, Hellmut G. Augustin, Peter Altevogt, Jochen Utikal, Zvi G. Fridlender, Viktor Umansky

**Affiliations:** 1 Skin Cancer Unit, German Cancer Research Center (DKFZ), 69120 Heidelberg, Germany; Christopher.Groth@medma.uni-heidelberg.de (C.G.); ninawinkler@live.de (N.W.); diester.klara96@web.de (K.D.); Rebekka.Weber@medma.uni-heidelberg.de (R.W.); ihor.arkhypov@gmail.com (I.A.); Samantha.Lasser@medma.uni-heidelberg.de (S.L.); Vera.Petrova@medma.uni-heidelberg.de (V.P.); p.altevogt@dkfz-heidelberg.de (P.A.); j.utikal@dkfz.de (J.U.); 2 Department of Dermatology, Venereology and Allergology, University Medical Center Mannheim, Ruprecht-Karl University of Heidelberg, 68167 Mannheim, Germany; 3 Faculty of Biosciences, Ruprecht-Karl University of Heidelberg, 69120 Heidelberg, Germany; 4 Mannheim Institute for Innate Immunoscience (MI3), Medical Faculty Mannheim, University of Heidelberg, 68167 Mannheim, Germany; 5 Institute of Pulmonary Medicine, Hebrew University Hadassah Medical Center, POB 12000, Jerusalem 9112001, Israel; ludovica.arpinati@mail.huji.ac.il (L.A.); meravsha@hadassah.org.il (M.E.S.); fridlender@hadassah.org.il (Z.G.F.); 6 European Center for Angioscience (ECAS), Medical Faculty Mannheim, Heidelberg University, 68167 Mannheim, Germany; n.gengenbacher@dkfz.de (N.G.); augustin@angioscience.de (H.G.A.); 7 Division of Vascular Oncology and Metastasis, German Cancer Research Center (DKFZ), 69120 Heidelberg, Germany

**Keywords:** PMN-MDSC, CXCR2, CXCL1, immunosuppression, melanoma, immunotherapy, metastasis, genetically engineered mouse model

## Abstract

**Simple Summary:**

Myeloid-derived suppressor cells (MDSC) represent a heterogeneous myeloid cell population that is expanded in tumor bearing hosts and substantially contributes to immunosuppression, representing thereby a valuable therapeutic target. Our study analyzes polymorphonuclear (PMN) and monocytic (M) MDSC subsets regarding their immunosuppressive capacity and recruitment mechanisms in murine melanoma. The immunosuppressive activity of both subsets was comparable. We identified the C-X-C Motif Chemokine Receptor (CXCR) 2/chemokine C-X-C motif ligand (CXCL) 1 axis as an important mediator of PMN-MDSC recruitment. Inhibition of CXCR2 resulted in a decreased infiltration of tumors with PMN-MDSC and increased survival of melanoma bearing mice. Furthermore, adjuvant treatment of mice with resected tumors reduced the infiltration of pre-metastatic sites with PMN-MDSC and the occurrence of distant metastasis. The decrease in PMN-MDSC infiltration was accompanied by an increase in natural killer (NK) cell frequency, suggesting an important role of PMN-MDSC in suppressing the NK cell-mediated anti-tumor response.

**Abstract:**

Background: Despite recent improvement in the treatment of malignant melanoma by immune-checkpoint inhibitors, the disease can progress due to an immunosuppressive tumor microenvironment (TME) mainly represented by myeloid-derived suppressor cells (MDSC). However, the relative contribution of the polymorphonuclear (PMN) and monocytic (M) MDSC subsets to melanoma progression is not clear. Here, we compared both subsets regarding their immunosuppressive capacity and recruitment mechanisms. Furthermore, we inhibited PMN-MDSC migration in vivo to determine its effect on tumor progression. Methods: Using the *RET* transgenic melanoma mouse model, we investigated the immunosuppressive function of MDSC subsets and chemokine receptor expression on these cells. The effect of CXCR2 inhibition on PMN-MDSC migration and tumor progression was studied in *RET* transgenic mice and in C57BL/6 mice after surgical resection of primary melanomas. Results: Immunosuppressive capacity of intratumoral M- and PMN-MDSC was comparable in melanoma bearing mice. Anti-CXCR2 therapy prolonged survival of these mice and decreased the occurrence of distant metastasis. Furthermore, this therapy reduced the infiltration of melanoma lesions and pre-metastatic sites with PMN-MDSC that was associated with the accumulation of natural killer (NK) cells. Conclusions: We provide evidence for the tumor^−^promoting properties of PMN-MDSC as well as for the anti-tumor effects upon their targeting in melanoma bearing mice.

## 1. Introduction

Cutaneous malignant melanoma is one of the deadliest tumors [1,2]. Primary melanomas are both able to metastasize regionally, to the lymph nodes (LN), or to distant sites including skin, lung, brain, liver and intestines [3]. Melanoma is currently treated with targeted therapy against mutations in the serine/threonine-protein kinase B-Raf (BRAF) or mitogen-activated protein kinase (MEK), which leads to tumor regression in up to 90% of patients [4]. Furthermore, the therapy with immune checkpoint inhibitors, in particular with antibodies against programmed cell death protein (PD)-1, resulted in a strong increase in the overall survival of advanced melanoma patients [5,6,7]. However, in many patients a durable tumor control was not possible. This could be attributed to a strong immunosuppressive tumor microenvironment mediated, in particular, by myeloid-derived suppressor cells (MDSC) that represent a heterogeneous myeloid cell population [8,9,10,11,12]. In mice, MDSC are characterized by the co-expression of CD11b and Gr1, consisting of two subunits, Ly6G and Ly6C [8]. Based on their expression levels, MDSC can be classified as Ly6G^+^Ly6C^lo^ polymorphonuclear (PMN)-MDSC and Ly6G^−^Ly6C^hi^ monocytic (M)-MDSC, which resemble morphological features of neutrophils and monocytes respectively [8].

The main feature of MDSC is a strong capacity to inhibit anti-tumor reactivity of effector T and NK cells by various mechanisms. These cells deplete L-arginine and cysteine that are important for T cell activation and proliferation [8,11,12]. They upregulate the expression of PD-L1, leading to T cell blockade via interaction with PD-1 expressed on effector T cells [8,13,14]. Moreover, reactive nitrogen species (RNS) such as nitric oxide (NO) secreted by MDSC results in T cell apoptosis or the nitration of TCR, reducing its signaling capacity [9,12,15,16]. High levels of MDSC-derived reactive oxygen species (ROS) impair clonal expansion and activation of T cells [8,10,11,17]. MDSC can also contribute to the production of immunosuppressive adenosine via increased expression of ectonucleotidases CD39 and CD73 [18,19]. In addition, MDSC were reported to induce the development of FoxP3^+^ regulatory T cells (Treg) in tumor bearing host [20] and to subvert tumor associated macrophages into M2 cells in a cell-contact dependent manner [21].

Being generated in the bone marrow, MDSC are recruited to the tumor site by a diverse set of chemokines. Whereas CCL2, CCL3, and CCL4 are critical for the recruitment of monocytic (M)-MDSC via the C-C chemokine receptor (CCR)2 [22], the ligands of the CXC- chemokine receptor (CXCR)2 CXCL1, −2, −3, −5, −6, and −7 primarily mediate PMN-MDSC migration [23]. In addition, CXCR4 and its ligand CXCL12 have been demonstrated to facilitate the accumulation of MDSC at the tumor site in different tumor models [24]. Importantly, MDSC have been reported to contribute to the process of metastasis [25]. In melanoma bearing mice, PMN-MDSC have been shown to produce hepatocyte growth factor (HGF) and transforming growth factor (TGF)-β that stimulate epithelial to mesenchymal transition (EMT) and dissemination of tumor cells [26]. Furthermore, MDSC support intravasation of tumor cells by secreting matrix metalloprotease (MMP)-2 and MMP-9, which remodel the basal membrane allowing the migration of neoplastic cells [27]. In addition, MDSC promote angiogenesis in pre-metastatic sites and the growth of micrometastases [28].

Using the *RET* transgenic mouse melanoma model that shows similarity to human melanoma [29,30], we demonstrated previously a critical role of MDSC in melanoma progression [31,32]. In this study, we systematically compared M- and PMN-MDSC subsets with respect to their immunosuppressive activity and mechanisms of migration to primary skin tumors and metastatic lesions of melanoma bearing mice. Furthermore, upon the selective inhibition of CXCR2 on PMN-MDSC we observed a reduction of their migration to the tumor lesions and pre-metastatic sites associated with prolonged survival and reduction of distant metastasis in melanoma bearing mice. Moreover, anti-CXCR2 therapy induced an accumulation of NK cells in the tumor microenvironment. Our findings highlight the unique role of PMN-MDSC in melanoma progression and the possibility to block PMN-MDSC migration to tumor lesions improving immunotherapy of melanoma patients.

## 2. Results

### 2.1. PMN-MDSC Subset Expands during Tumor Progression

**Figure 1:**
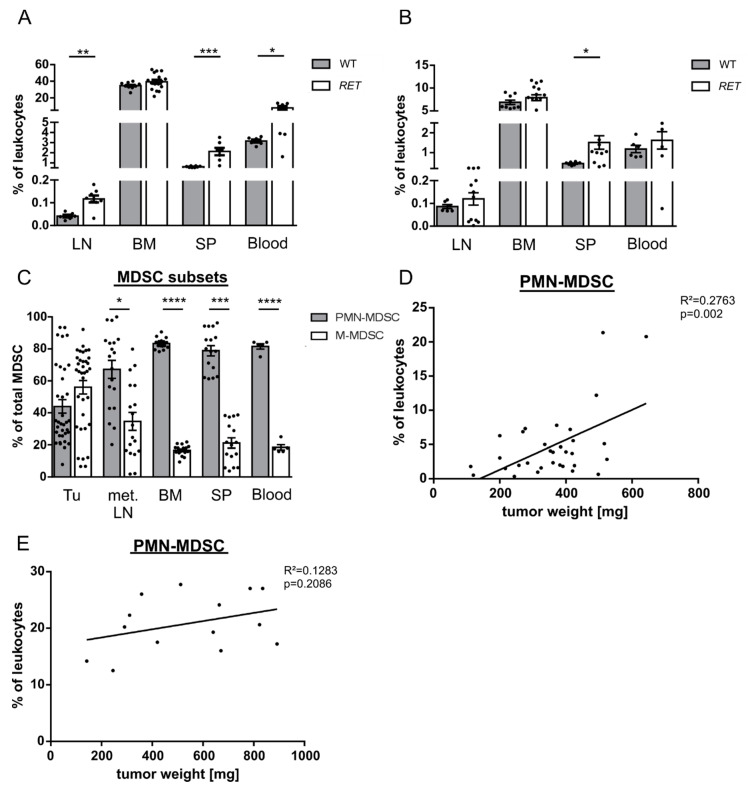
Myeloid-derived suppressor cells (MDSC) subpopulations in mice bearing melanoma or mesothelioma and their non-suppressive counterparts in healthy C57BL/6 mice. CD11b^+^Ly6G^+^Ly6C^lo^ polymorphonuclear (PMN)-MDSC (**A**) and CD11b^+^Ly6G^−^Ly6C^hi^ M-MDSC (**B**) of *RET* transgenic mice and their non-suppressive counterparts in C57BL/6 mice (**A**,**B**) were analyzed in lymph nodes (LN), bone marrow (BM), spleen (SP), and peripheral blood by flow cytometry. Data are presented as the percentage of PMN- or monocytic (M)-MDSC from tumor-bearing mice or their non-suppressive counterparts from healthy mice within CD45^+^ leukocytes (mean ± SEM; *n* = 6–16 mice/group). All samples were collected from tumor bearing mice in different time points between the appearance of macroscopically visible tumors and the appearance of abortion criteria. (**C**) PMN- and M-MDSC were measured in primary tumors (TU), metastatic LN (met. LN), BM, SP, and peripheral blood of *RET* transgenic mice and expressed as the percentage of PMN- or M-MDSC within total MDSC (mean ± SEM; *n* = 5–35 mice/group). The weight of each melanoma (**D**) or mesothelioma samples (**E**) in mg was plotted against the percentage of PMN-MDSC among CD45^+^ leukocytes in tumors from respective mice (*n* = 14–32). The correlation was evaluated by a linear regression analysis. * *p* < 0.05, ** *p* < 0.01, *** *p* < 0.001, **** *p* < 0.0001.

We measured the frequency of M- and PMN-MDSC in melanoma lesions (primary skin tumor and metastatic lymph nodes (LN)), the peripheral blood, spleen and bone marrow (BM) by flow cytometry, using the *RET* transgenic mouse model, which mimics the human situation (Figure S1). The frequency of PMN-MDSC was found to be significantly higher in LN, spleen and peripheral blood of tumor bearing mice compared to their non-suppressive counterpart in these organs from healthy animals (Figure 1A). A similar trend was observed for M-MDSC, which was significant for spleen (Figure 1B). Moreover, PMN-MDSC represented the dominant subset among the total MDSC population in all studied organs except primary skin tumors, where a tendency for M-MDSC to be present at higher frequencies was detected (Figure 1C). The accumulation of tumor infiltrating PMN-MDSC was significantly correlated with the elevated weight of primary tumors that is considered as an indicator of tumor progression (Figure 1D). Interestingly, the infiltration of skin tumors with M-MDSC failed to be dependent on melanoma progression (Figure S2A). Recent studies with injected AB12 mesothelioma cells suggested a critical role of PMN-MDSC in contrast to M-MDSC in suppressing the anti-tumor response [33]. To corroborate our observations, we analyzed the tumor infiltrating MDSC subpopulations in a mouse mesothelioma model and found similar tendencies regarding the enrichment of PMN- (Figure 1E) and M-MDSC (Figure S2B) as in the melanoma microenvironment.

### 2.2. M-MDSC and PMN-MDSC Display a Comparable Immunosuppressive Phenotype in Primary Melanomas

**Figure 2:**
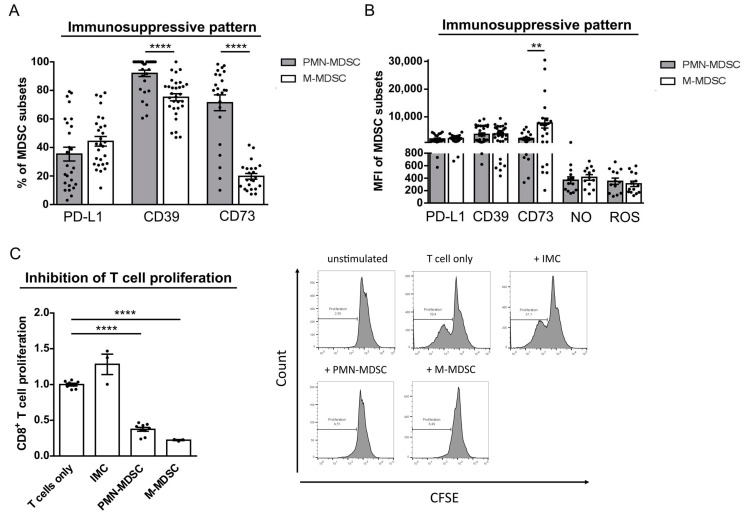
Immunosuppressive activity of MDSC subsets in melanoma bearing *RET* transgenic mice. (**A**,**B**) The expression of PD-L1, CD39 and CD73 as well as the production of NO and ROS was measured in PMN- and M-MDSC isolated from skin tumors of RET transgenic mice. The results are presented as the percentage of PD-L1^+^, CD39^+^ or CD73^+^ cells within respective MDSC subpopulations ((**A**), mean ± SEM; *n* = 13–29 mice/group) or the level of their expression as well as of the NO and ROS production measured as mean fluorescence intensity (MFI) (**B**) For staining of CD73 and PD-L1 isotype controls were used while CD39, NO, and ROS expression was determined using up to fluorescence minus four (FM4) controls. (**C**) Inhibition of CD8^+^ T cell proliferation by PMN- or M-MDSCs and representative histograms for the corresponding FACS stainings. T cells stimulated with anti-CD3 and anti-CD28 antibodies were cultured for 96 h alone or together with CD11b^+^Gr1^+^ immature myeloid cells (IMC) from the BM of healthy mice or with tumor PMN- or M-MDSC from skin melanoma at the T cell:MDSC ratio 1:2. Proliferation was analyzed by flow cytometry. Data are normalized to the T cells only control (mean ± SEM; *n* = 3–9 mice/group). ** *p* < 0.01, **** *p* < 0.0001.

To compare the immunosuppressive pattern of PMN-MDSC and M-MDSC, we determined the frequency of PD-L1, CD39, and CD73 and the level of their expression (detected as mean fluorescence intensity, MFI) in skin tumors of *RET* transgenic mice. On average, 40% of both MDSC subsets expressed PD-L1 showing a similar MFI level (Figure 2A,B and Figure S3). In contrast, the frequency of CD39^+^ and especially CD73^+^ cells was significantly higher within PMN- than M-MDSC (Figure 2A and Figure S3). Although we found no difference in the level of CD39 expression intensity on both subsets, the expression level of CD73 was significantly higher in M-MDSC (Figure 2B). Furthermore, both MDSC subsets produced other immunosuppressive mediators such as NO and ROS to a comparable degree (Figure 2B). Finally, we tested an immunosuppressive activity of tumor infiltrating PMN- and M-MDSC isolated from melanoma bearing *RET* transgenic mice. Both subsets demonstrated a strong capability to suppress the proliferation of activated CD8^+^ T cells in vitro to a similar extent (Figure 2C).

### 2.3. PMN-MDSC Selectively Express CXCR2

**Figure 3:**
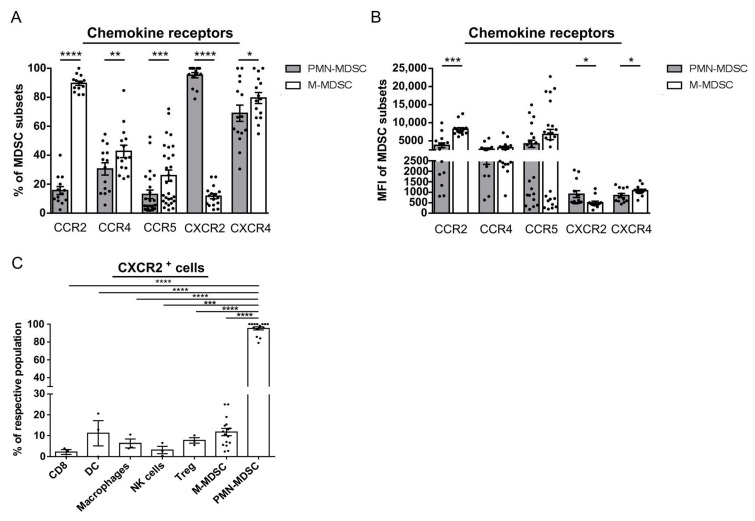
Chemokine receptor expression on melanoma infiltrating immune cells from RET transgenic mice. Expression of CCR2, CCR4, CCR5, CXCR2, and CXCR4 on tumor infiltrating PMN- and M-MDSC from RET transgenic mice was detected by flow cytometry. Data are shown as the percentage cells positive for chemokine receptors among respective MDSC subsets (**A**) or the level of their expression measured as MFI (**B**) (mean ± SEM; *n* = 12–29 mice/group). (**C**) Expression of CXCR2 on intratumoral CD8^+^ T cells, CD11c^+^ dendritic cells (DC), F4/80^+^ macrophages, CD3^−^NK1.1^+^ natural killer (NK) cells, CD4^+^FoxP3^+^CD25^+^ regulatory T cells (Treg), M-MDSC and PMN-MDSC was detected by flow cytometry and are presented as the percentage of CXCR2^+^ cells within or the respective immune cell population (mean ± SEM; *n* = 3–15 mice/group). * *p* < 0.05, ** *p* < 0.01, *** *p* < 0.001, **** *p* < 0.0001.

Next, we studied the expression of CCR2, CCR4, CCR5, CXCR2, and CXCR4, which were described to promote MDSC recruitment. The majority of chemokine receptors was expressed on both subsets of tumor infiltrating MDSC. However, M-MDSC displayed a significantly higher level of CCR2, CCR4, CCR5, and CXCR4 than PMN-MDSC (Figure 3A). In contrast, CXCR2 was almost exclusively expressed on PMN-MDSC (Figure 3A). CCR2 was present on 80–90% of M-MDSC, whereas its expression was found only on less than 20% of PMN-MDSC. In addition, CCR2 expression level was significantly lower on PMN-MDSC than on M-MDSC (Figure 3B). Significantly higher percentages of M-MDSC expressed CCR4 and CCR5 (Figure 3A). Additionally, more M-MDSC showed CXCR4 expression with a significant elevation of its intensity (Figure 3A,B). Interestingly, almost all PMN-MDSC were found to express CXCR2, while only 10% of M-MDSC showed its expression with significantly lower intensity than their PMN counterpart (Figure 3A,B). Comparing the expression of CXCR2 on other melanoma infiltrating immune cells, including CD8^+^ T cells, dendritic cells (DC), macrophages, NK cells, Treg, and M-MDSC, we found it almost ubiquitously present on PMN-MDSC but only to a very low extent on other cell populations (Figure 3C). In contrast to our findings in the melanoma mouse model, only very few PMN-MDSC infiltrating mesothelioma expressed CXCR2 (Figure S4).

### 2.4. CXCL1 Mediates Migration of PMN-MDSC via CXCL1 In Vitro

**Figure 4:**
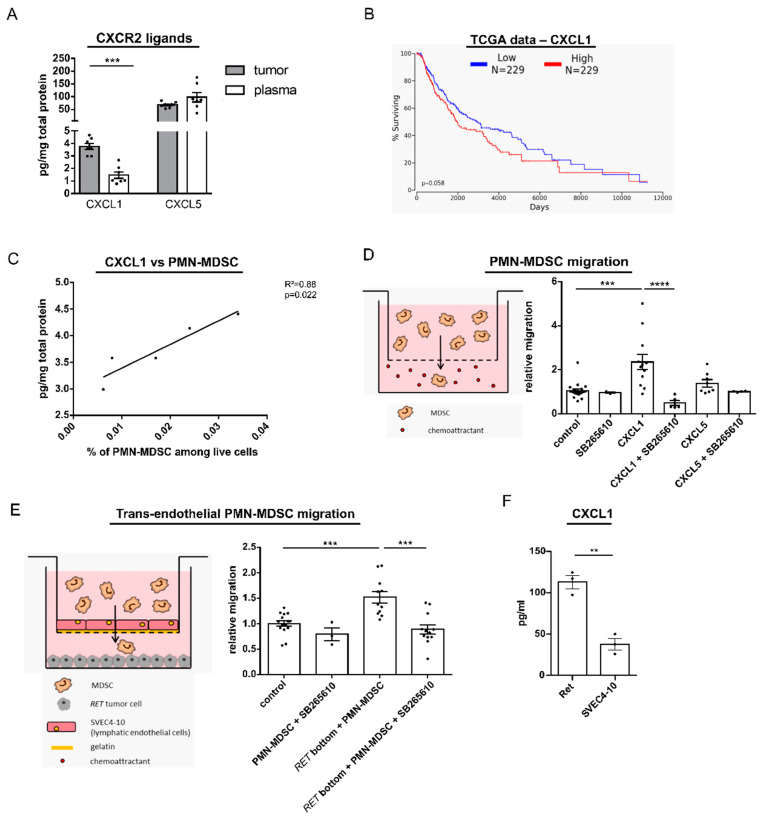
Characterization of CXCR2/CXCL1 and CXCR2/CXCL5 interaction in melanoma. (**A**) Concentrations of CXCL1 and CXCL5 were determined in plasma and lysates of skin tumors of RET transgenic mice by bio-plex analysis and expressed as pg/mg total protein (mean ± SEM; *n* = 7–8). (**B**) Overall survival analysis of melanoma patients (*n* = 458) stratified into those with low and high CXCL1 expression (lower and upper percentile 50). (**C**) The intratumoral levels of CXCL1 expressed as pg/mg protein were plotted against the percentage of tumor infiltrating PMN-MDSC among total live cells from respective mice (*n* = 5). The correlation was evaluated by a linear regression analysis. (**D**) Spontaneous migration (control) and migration towards CXCL1 (100 ng/mL) or CXCL5 (100 ng/mL) of PMN-MDSC from the BM of tumor bearing RET transgenic mice was determined in the presence and absence of the CXCR2 inhibitor SB265610 (100 nM) after the incubation for 24 h in a transwell assay. Data are normalized to the spontaneous migration of PMN-MDSC (control) (mean ± SEM; *n* = 3–19). (**E**) Spontaneous migration of PMN-MDSC from the BM of tumor bearing transgenic mice and their migration towards Ret tumor cells through SVEC4-10 lymphatic endothelial cells were determined in the presence and absence of the CXCR2 inhibitor SB265610 (100 nM) in a transwell assay after 24 h. Data are normalized to the spontaneous migration of PMN-MDSC (control) (mean ± SEM; *n* = 3–15). (**F**) Production of CXCL1 by Ret tumor cells and SVEC4-10 endothelial cells determined by Enzyme-Linked Immunosorbent Assay (ELISA) in cell culture supernatant (mean ± SEM; *n* = 3). ** *p* < 0.01, *** *p* < 0.001, **** *p* < 0.0001.

Since CXCR2 was found to be expressed almost exclusively on PMN-MDSC, we aimed to further investigate its functional relevance in melanoma. Both ligands of CXCR2, CXCL1, and CXCL5 were detected in tumors and plasma of RET transgenic mice (Figure 4A). Importantly, CXCL1 concentration in tumor lysates was found to be significantly higher than in plasma from corresponding mice, whereas the level of CXCL5 did not significantly differ in tumor and plasma samples. To evaluate the importance of CXCL1 in the recruitment of immunosuppressive cells in human setting in relation to the clinical outcome, we analyzed TCGA data of 458 melanoma patients. The results showed a strong correlation of elevated intratumoral concentrations of CXCL1 with decreased overall survival of melanoma patients (Figure 4B). In contrast, such correlation was not found for CXCL5 levels (Figure S5A). In addition, increased intratumoral CXCL1 level in RET transgenic mice, in contrast to CXCL5, significantly correlated with the accumulation of melanoma infiltrating PMN-MDSC (Figure 4C and Figure S5B).

Based on these observations, we focused on the role of CXCR2/CXCL1 axis in PMN-MDSC recruitment in our further experiments. In vitro transwell migration assays demonstrated a strong migration of BM-derived PMN-MDSC from melanoma bearing mice towards a gradient of the CXCL1 concentration (Figure 4D). This effect was efficiently blocked by the small molecule inhibitor of CXCR2, SB265610 (Figure 4D). Furthermore, we found a tendency for migration of PMN-MDSC stimulated by CXCL5, although it was not statistically significant (Figure 4D). In contrast to PMN-MDSC, M-MDSC failed to migrate towards CXCL1 or CXCL5 in vitro (Figure S6). To investigate if melanoma cells are capable of recruiting PMN-MDSC via CXCR2 from the periphery, we cultured Ret melanoma cells in the bottom compartment of a transwell and determined the number of migrated PMN-MDSC towards Ret cells through SVEC4-10 lymphatic endothelial cells (Figure 4E). The presence of tumor cells significantly stimulated the migration of PMN-MDSC, which was completely abrogated when the CXCR2 inhibitor was added to the culture (Figure 4E,). Importantly, Ret melanoma cells were found to produce significantly higher amounts of CXCL1 than SVEC4-10 lymphatic endothelial cells (Figure 4F).

### 2.5. CXCR2 Inhibition Increases the Survival of Melanoma Bearing RET Transgenic Mice

**Figure 5:**
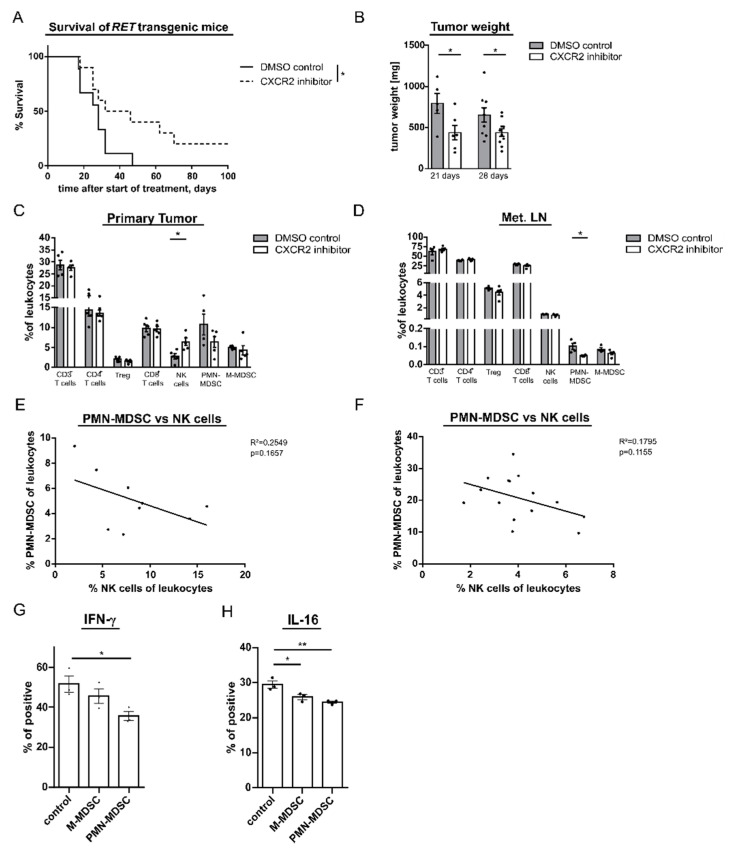
Effect of anti-CXCR2 therapy on mouse survival and on tumor infiltrating immune cells. RET transgenic melanoma bearing mice were injected i.p. with the CXCR2 inhibitor SB265610 (2 mg/kg) or the control DMSO solution (control group) twice a week for four weeks. (**A**) Survival of mice (9–10 mice/group) is shown as a Kaplan–Meier curve. (**B**) Weight of tumors from mice treated with SB265610 or the control injection was measured at day 21 and 28 after the treatment initiation in two independent experiments. Results are shown in mg. One day after the last injection, cells infiltrating primary skin tumors (**C**) and metastatic LN (**D**) were measured by flow cytometry. Data are presented as the percentage of CD3^+^, CD4^+^ and CD8^+^ T cells, CD4^+^CD25^+^FoxP3^+^ Treg, CD3^−^NK1.1^+^ NK cells, PMN- and M-MDSC within CD45^+^ leukocytes (mean ± SEM; 4–5 mice/group). The percentage of PMN-MDSC among CD45^+^ leukocytes infiltrating primary melanoma (**E**) or mesothelioma (**F**) were plotted against the percentage of tumor infiltrating NK cells within CD45^+^ leukocytes from respective mice (*n* = 9–15). The correlation was evaluated by a linear regression analysis. Mouse NK cells were cultured with in vitro differentiated PMN- or M-MDSC at a ratio of 1:2. Frequency of IFN-γ (**G**) or IL-16 producing NK cells (**H**) was determined by flow cytometry after 4 h of coculture. NK cells cultured alone (control) served as a negative control. Data are shown as the percentage of IFN-γ^+^ or IL-16^+^ NK cells within total NK cells (mean ± SEM; *n* = 3). * *p* < 0.05, ** *p* < 0.01.

Next, we tested the effect of CXCR2 inhibition on melanoma progression in vivo. Tumor bearing RET transgenic mice were treated with the CXCR2 inhibitor SB265610, which led to a significant survival benefit as compared to mice treated with the DMSO solution (control group) (Figure 5A). The observed anti-tumor effect was indicated also by a decrease in tumor weight upon anti-CXCR2 therapy (Figure 5B). In contrast to melanoma, anti-CXCR2 therapy did not reduce tumor weight in the mesothelioma mouse model (Figure S7). Since CXCR2 may also stimulate tumor endothelial cells [34], we measured the expression of intratumoral CD31 and found no changes upon the therapy (Figure S8), indicating the absence of effect on tumor vascularization.

To determine the mechanisms leading to the prolonged survival under the anti-CXCR2 therapy, we analyzed tumor infiltrating MSDC, T, and NK cells. We found that the frequency of PMN-MDSC was significantly decreased in metastatic LN and showed a tendency for the reduction in primary skin tumors from mice treated with the CXCR2 inhibitor as compared to control group (Figure 5C,D). Interestingly, the frequency of intratumoral CD3^+^ T cells, CD8^+^ T cells, total CD4^+^ T cells, Treg, and M-MDSC remained unchanged upon the therapy in both melanoma lesions. However, we observed a significant increase in the frequency of NK cells infiltrating skin tumors (Figure 5C). Moreover, the accumulation of these cells tended to correlate with the decrease in the frequency of PMN-MDSC both in skin melanoma and mesothelioma (Figure 5E,F). Interestingly, PMN-MDSC frequencies in the BM and peripheral blood were not significantly altered upon the therapy, indicating that anti-CXCR2 therapy could inhibit PMN-MDSC migration to the tumor without affecting their amounts at the systemic level (Figure S9). To investigate if PMN-MDSC are able to reduce the activation of NK cells, we cultured pre-activated NK cells with PMN- or M-MDSC in vitro. It was found that PMN-MDSC significantly reduced the production of IFN-γ and IL-16, indicating an inhibition of NK cell activity (Figure 5G,H). Interestingly, the suppressive effect of M-MDSC was less prominent under these conditions than this effect of PMN-MDSC (Figure 5G,H).

### 2.6. CXCR2 Inhibition Reduces Development of Metastases

**Figure 6:**
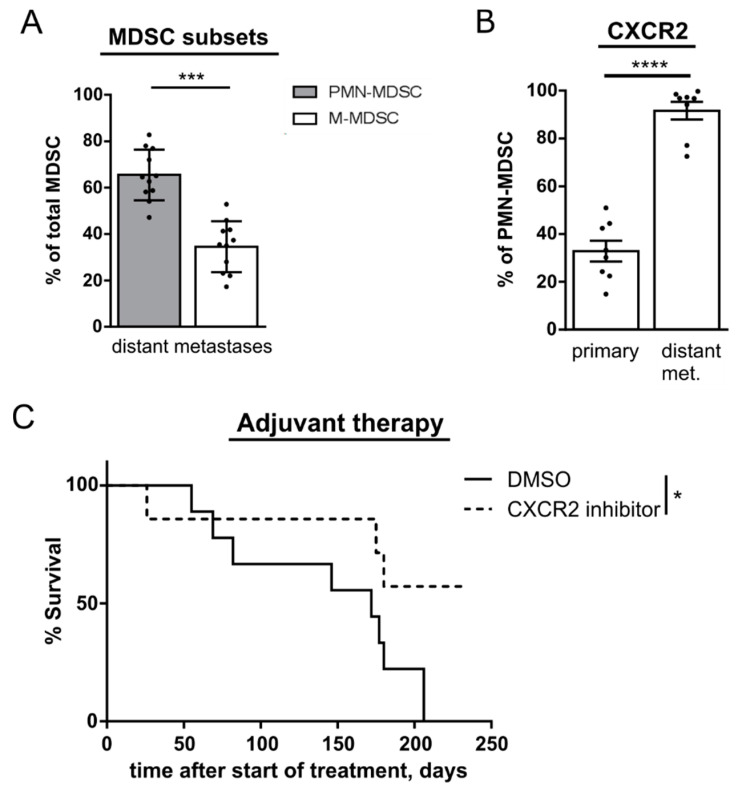
Accumulation of PMN-MDSC in melanoma metastasis and effect of adjuvant anti-CXCR2 therapy on mouse survival. C57BL/6 mice were transplanted intradermally with the fragments of primary tumors from RET transgenic mice followed by tumor resection at day 21 after transplantation. (**A**) PMN- and M-MDSC were analyzed in distant metastases (lungs and liver) of melanoma bearing mice by flow cytometry. Data are presented as the percentage of PMN- or M-MDSC among total MDSC (mean ± SEM; *n* = 11). (**B**) PMN-MDSC in primary skin tumors and distant metastases. Data are shown as the percentage of these cells among total MDSC (mean ± SEM; *n* = 8). (**C**) At day 7 after tumor resection, mice were injected i.p. with the CXCR2 inhibitor SB265610 (2 mg/kg) or the control DMSO solution (control group) twice a week for six weeks. Survival of mice (7–10 mice/group) is shown as Kaplan–Meier curves. * *p* < 0.05, *** *p* < 0.001, **** *p* < 0.0001.

Having demonstrated that anti-CXCR2 therapy reduced primary tumor weight and prolonged mouse survival, we studied the effect of CXCR2 inhibition on the metastatic process in an adjuvant setting. Fragments of primary tumors from RET transgenic mice were transplanted intradermally into normal C57BL/6 mice. After 21 days, primary tumors were resected, leading to the formation of metastases in LN and distant organs (primarily in liver and lungs) 4 months later. We found that PMN-MDSC were the dominant subset among MDSC in distant metastases (Figure 6A). In addition, CXCR2 expression was significantly more frequent on PMN-MSDC infiltrating distant metastases as compared to those accumulated in primary skin tumors (Figure 6B). Importantly, the treatment with CXCR2 inhibitor significantly prolonged the survival of mice with resected primary melanomas (Figure 6C).

**Figure 7:**
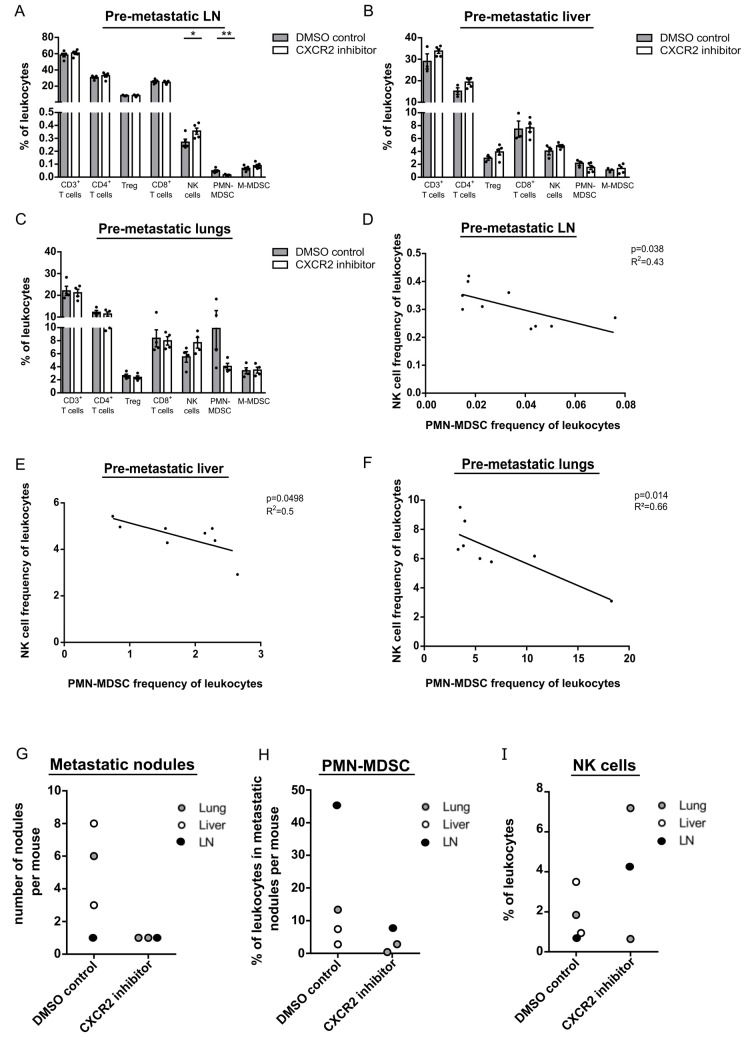
Anti-CXCR2 therapy impairs PMN-MDSC migration to pre-metastatic sites and metastasis in LN, liver, and lungs. C57BL/6 mice were transplanted intradermally with the fragments of primary tumors from RET transgenic mice followed by tumor resection at day 21 after transplantation. At day 7 after resection, mice were injected i.p. with the CXCR2 inhibitor SB265610 (2 mg/kg) or the control DMSO solution (control group) twice a week for four weeks. (**A**–**C**) One day after the last injection, CD3^+^, CD4^+^, and CD8^+^ T cells, Treg, NK cells, PMN- and M-MDSC were measured in pre-metastatic LN (**A**), liver (**B**) and lungs (**C**) by flow cytometry. Data are presented as the percentage of respective subpopulations within CD45^+^ leukocytes (mean ± SEM; 3–5 mice/group). The percentage of PMN-MDSC among CD45^+^ leukocytes infiltrating pre-metastatic LN (**D**), liver (**E**), or lungs (**F**) were plotted against the percentage of tumor infiltrating NK cells within CD45^+^ leukocytes in these organs from respective mice (*n* = 8–10). The correlation was evaluated by a linear regression analysis. One day after the last injection, macroscopic metastatic nodules in LN, lungs, and liver were counted (**G**). Data are shown as number of nodules per mouse. The frequency of PMN-MDSC (**H**) and NK cells (**I**) infiltrating metastatic nodules in LN, lungs, and livers were measured in each nodule per mouse by flow cytometry and presented as the percentage of respective cell populations among CD45^+^ leukocytes. * *p* < 0.05, ** *p* < 0.01.

We, therefore, hypothesized that we could impair the formation of pre-metastatic sites in LN, lungs and liver by inhibiting the migration of PMN-MDSC, causing thereby a delayed initiation of metastasis. Indeed, the frequency of PMN-MDSC was significantly reduced in pre-metastatic LN of treated mice as compared to the control group, whereas NK cell frequency was increased (Figure 7A). Moreover, the same trend of changes in PMN-MDSC and NK cell infiltration (although not statistically significant) was detected in pre-metastatic liver and lungs (Figure 7B,C). In addition, the decreased frequency of PMN-MDSC in treated mice significantly correlated with an augmented NK cell frequency in pre-metastatic LN, liver, and lungs (Figure 7D–F).

Observing further the development of metastases in mice receiving anti-CXCR2 therapy, we found that they had less macroscopic metastatic nodules in lungs and liver than in mice without the inhibition of PMN-MDSC recruitment (Figure 7G). Furthermore, these metastatic nodules were characterized by a diminished infiltration with PMN-MDSC and by an accumulation of NK cells (Figure 7H,I).

## 3. Discussion

In this study, we compared PMN- and M-MDSC subsets during melanoma progression since these MDSC subsets could partially rely on different mechanisms of their immunosuppressive capacity [35]. We found that PMN-MDSC represent the majority of cells within the total population of MDSC in metastatic LN, BM, spleen, and peripheral blood of tumor bearing *RET* transgenic mice. Interestingly, the accumulation of melanoma infiltrating MDSC in this transgenic mouse model was primarily caused by an enrichment of PMN-MDSC, while the frequency of M-MDSC remained constant during tumor progression. This indicates that PMN-MDSC play a dominant role in immunosuppressive melanoma microenvironment, especially at later stages of tumor progression. The observed enrichment of intratumoral PMN-MDSC may also occur in other tumor types since we observed similar results in murine mesotheliomas. Interestingly, a correlation of PMN-MDSC accumulation with increased tumor size has been recently reported for pancreatic cancer [36]. PMN-MDSC have been characterized as non-proliferating cells with a short half-life span, whereas M-MDSC can proliferate and further differentiate into tumor associated macrophages [8,9,11,37]. Therefore, it is conceivable that MDSC may be recruited constantly to the tumor site and that the observed preferential accumulation of PMN-MDSC is mediated primarily by an increased production of factors involved in PMN-MDSC recruitment. In addition, it has been recently reported that monocyte-like precursors of granulocytes were strongly expanded and differentiated to PMN-MDSC in tumor bearing mice that could explain an accumulation of this MDSC subset [38].

Analyzing the expression of immunosuppressive molecules, we found that both MDSC subsets infiltrating primary skin tumors showed a similar expression of PD-L1 that was shown to be important for their immunosuppressive effect on T cells [8,12,13,14]. However, significantly larger proportion of PMN-MDSC displayed ectonucleotidase CD39 and CD73 than that of M-MDSC. Since CD39 and CD73 can convert ATP into adenosine that inhibits T cell functions [39], their predominant expression on PMN-MDSC suggests a more important role of adenosine in immunosuppressive activity of this MDSC subset. This finding is of relevance since CD73 expression has been reported to be associated with the formation of LN metastases and tumor progression in different cancer entities [40]. In addition, high CD39/CD73 co-expression on circulating MDSC has been shown to be associated with the progression of non-small-cell lung carcinoma (NSCLC) [19]. Although it was previously demonstrated that M-MDSC produced more NO, whereas PMN-MDSC showed higher ROS production [41], we did not detect significant differences in the production of these immunosuppressive factors by both MDSC subsets. Comparing the immunosuppressive capacity of PMN-MDSC and M-MDSC isolated from the same skin melanomas, we observed that both subpopulations significantly inhibited the proliferation of activated T cells and to a similar degree. Although a higher immunosuppressive capacity of PMN-MDSC could be expected due to increased expression of CD39 and CD73, it is likely that other mechanisms that were not evaluated for M-MDSC in our study may compensate lower expression of adenosine producing ectonucleotidases. For example, M-MDSC have been shown to exert their immunosuppressive capacity via the immune-regulatory TIM-3/galectin-9 pathway that could mediate the resistance to PD-1 blockade in NSCLC patients [42]. Therefore, the relative contribution of each MDSC subset into immunosuppression might be dependent on cancer type or the stage of tumor progression tumor.

We demonstrated that intratumoral M-MDSC express higher level of CCR2, CCR4, and CCR5. This is in line with reports that identified CCR2 ligand CCL2 and CCR5 ligand CCL5 as the main factors, driving M-MDSC migration to the tumor in vivo [43]. In contrast, another study suggested that CCR5 is primarily important for the recruitment of PMN-MDSC to the tumor site [44]. However, findings presented here and our previous publication [32] indicated a relevance of this receptor for both MDSC subpopulations. Furthermore, we observed that both MDSC subsets expressed CXCR4 with a small but significant prevalence for M-MDSC. Interaction between CXCR4 and its ligand CXCL12 was described to mediate the MDSC trafficking in mouse models of breast cancer [45] and hepatic carcinoma [46].

However, we found that CXCR2 was the only studied chemokine receptor that was expressed almost on all PMN-MDSC, showing a very low expression on M-MDSC and other immune cells. These data are in line with other findings on the role of CXCR2 in the recruitment of PMN-MDSC [47]. Importantly, we detected significantly elevated concentrations of the CXCR2 ligand CXCL1 in skin melanoma lysates as compared to plasma. In addition, increased intratumoral levels of CXCL1 significantly correlated with decreased survival of melanoma patients and increased frequencies of PMN-MDSC. Furthermore, blocking of CXCR2 in vitro completely abolished the recruitment of PMN-MDSC by CXCL1-secreting Ret melanoma cells. Taken together, these data support a prominent role of CXCR2/CXCL1 axis in the recruitment of PMN-MDSC in melanoma, indicating this receptor as an ideal target for blocking migration of these cells without impairing the trafficking of tumor-reactive effector immune cells.

Using the *RET* transgenic mouse melanoma model, which resembles melanoma development in patients [29,30], we found a significant reduction of PMN-MDSC in the tumor microenvironment that was associated with a survival benefit upon the therapy with the CXCR2 inhibitor. Interestingly, the treated mice displayed no enhancement of CD8^+^ T cell infiltration but rather an enrichment of NK cells in tumors. Importantly, NK cells were previously reported to be a target of PMN-MDSC immunosuppressive effects in different malignancies [48]. Furthermore, NK cells have been demonstrated to be enriched in tumors of mice with melanoma and lung carcinoma exposed to the exercise training [49]. Such increase in NK cell infiltration resulted in reduced primary tumor growth and metastasis. In addition, an increased ratio of NK cells to LOX-1^+^ PMN-MDSC predicted positive therapy response of patients with non-small cell lung carcinoma, indicating an important role of NK cell inhibition by PMN-MDSC in tumor progression [50]. Although CXCR2 was reported to be expressed also on the vascular endothelium, contributing to tumor angiogenesis [34], we failed to observe any changes in the frequency of CD31^+^ tumor infiltrating endothelial cells upon CXCR2 inhibition. Therefore, an anti-tumor effect of CXCR2 inhibition in *RET* transgenic mice could be mediated by reduced migration of CXCR2^+^ PMN-MDSC to the tumor, resulting in the accumulation of NK cells. Interestingly, we found that PMN-MDSC infiltrating mesothelioma showed much lower level of CXCR2 expression than in melanoma that might explain the lack of anti-tumor effect of CXCR2 blockade, indicating that the beneficial effect of such therapy might be successful not in all cancer types. Although we observed CXCR2 expression on 30% of M-MDSC, anti-CXCR2 therapy failed to reduce the recruitment of M-MDSC in mesothelioma. This may be due to the lower frequency of CXCR2^+^ M-MDSC (30%) than that of CXCR2^+^ PMN-MDSC infiltrating melanoma. In addition, anti-CXCR2 treatment in mesothelioma was shorter and less frequent than in melanoma due to a rapid mesothelioma progression that could be insufficient to decrease the tumor infiltration with CXCR2^+^ cells.

Analyzing the involvement of PMN-MDSC in the metastatic process, we found that their frequency in distant metastases (in liver and lungs) was significantly elevated as compared to their monocytic counterpart. Moreover, a significantly higher expression of CXCR2 in PMN-MDSC infiltrating distant metastases indicated a high relevance of this receptor for migration of this MDSC subset to metastatic organs. Indeed, we observed that the application of the CXCR2 inhibitor in the adjuvant setting after resection of primary melanomas diminished the number of macroscopic metastatic nodules in lungs and liver and markedly prolonged mouse survival. Importantly, an adjuvant anti-CXCR2 therapy reduced PMN-MDSC and increased NK cell frequencies in the pre-metastatic sites in LN, lungs and liver. Similar changes were found for these cells infiltrating macroscopic metastatic nodules. Such observations are in line with a recent publication reporting that the CXCR2 inhibitor could significantly decrease the trafficking of MDSC in metastatic LN associated with an enhanced accumulation of NK cells in orthotopic breast cancer mouse model [51]. Interestingly, other reports on a mouse model of pancreatic ductal adenocarcinoma demonstrated that the treatment with the CXCR2 inhibitor caused an improved response to chemotherapy with 5-Fluoruracil associated with a decrease in PMN-MDSC and an increase in CD8^+^ T cells [52] or the protection from liver metastasis linked to an increase in CD3^+^ T cells [53].

Therefore, by targeting the CXCR2 receptor we were able to impair PMN-MDSC migration to the melanoma microenvironment. This led to the inhibition of primary tumor growth and metastasis in LN and distant organs (liver and lungs) as well as to the prolongation of survival of melanoma bearing mice. Reduced melanoma infiltration with PMN-MSDC was found to be correlated with increased amounts of intratumoral NK cells. Importantly, NK cells demonstrated higher sensitivity to the immunosuppression mediated by PMN-MDSC as compared to M-MDSC, indicating a critical impact of PMN-MDSC in the inhibition of anti-tumor functions of NK cells.

## 4. Materials and Methods

### 4.1. Mice

C57BL/6 mice, expressing the human *RET* oncogene in melanocytes under the mouse metallothionein-I promotor-enhancer [29] were provided by Dr. I. Nakashima (Chubu University, Aichi, Japan). Mice were kept under specified pathogen-free conditions in the animal facility of the University Medical Center (Mannheim, Germany). Non-transgenic littermates were used as normal C57BL/6 mice. All samples were collected from tumor bearing mice in different time points between the appearance of macroscopically visible tumors and the appearance of abortion criteria. To study mesotheliomas, 5–6 weeks old female Balb/c mice were purchased from Harlan Laboratories (Jerusalem, Israel). Mice were housed under specific pathogen-free conditions at the Hebrew University School of Medicine Animal Resource Center. The protocols were approved by the animal care and use Committee (IACUC) of the Hebrew University School of Medicine. In all experiments, animals were euthanized before surgery.

### 4.2. Cell Culture

Ret melanoma cell line was established from skin melanomas isolated from *RET* transgenic mice [54]. Mouse lymphatic endothelial cell line SVEC4-10 was established from endothelial cells from axillary LN vessels [55]. Mouse mesothelioma AB12 cell line was purchased from the CellBank Australia. All cell lines were cultured in Dulbecco’s Modified Eagle Medium (DMEM) (PAN-Biotech GmbH, Aidenbach, Germany) with 10% heat-inactivated FBS (Merck KGaA, Darmstadt, Germany) and 1% penicillin/streptomycin (Thermo Fisher, Waltham, MA, USA) at 37 °C and 5% CO_2_. All cell lines were regularly tested for the absence of mycoplasma contamination. The CXCR2 inhibitor SB265610 (100 nM) (Research And Diagnostic Systems, Inc., Minneapolis, MN, USA) and the CXC chemokines CXCL1 (100 ng/mL) and CXCL5 (100 ng/mL) (both from PeproTech, Inc., Rocky Hill, NJ, USA) were used for the treatment in vitro.

### 4.3. Isolation of Immature Myeloid Cells and MDSC Subsets

We purified CD11b^+^Gr1^+^ immature myeloid cells from normal mouse bone marrow as well as Ly6G^+^Ly6C^lo^ PMN-MDSC and Ly6G^−^Ly6C^hi^ M-MDSC from tumors and the bone marrow of *RET* transgenic mice using magnetic-activated cell sorting (MACS^®^, Miltenyi Biotec B.V. & Co. KG, Bergisch Gladbach, Germany, Cat. 130-094-538, the purity of isolated cells was more than 85%) according to the manufacturer’s instructions. Mouse tumors were treated with collagenase (1 mg/mL) and DNase (10 µg/mL, both Sigma-Aldrich, St. Louis, MO, USA) digested and filtered through a 100 µm cell strainer (Corning Inc., Corning, NY, USA). To enrich leukocytes, single cell suspension was centrifuged over Histopaque^®^ (1.119 g/mL, Thermo Fisher Scientific, Waltham, MA, USA).

### 4.4. Flow Cytometry

Cells were incubated with 7AAD or fixable viability dye 700 and with FcR Blocking Reagent (all BD biosciences, Franklin Laker, NJ, USA) followed by the staining with various fluorescence-labeled antibodies (Table S1). For intracellular staining, cells were fixed and permeabilized with the eBioscience^TM^ Foxp3/Transcription Factor Staining Buffer Set (Thermo Fisher Scientific, Waltham, MA, USA) according to the manufacturer’s instructions. To measure NO and ROS production, we used hROS Detection Kit (Cell Technology Inc., Minneapolis, MN, USA) and diaminofluorescein-FM diacetate (DAF-FM DA, Cayman Chemical, Ann Arbor, MI, USA) respectively. Acquisition was performed by ten-color flow cytometry by BD FACSLyric^TM^ with FACSuite^TM^ software (BD Biosciences, Franklin Laker, NJ, USA). The results were analyzed by FlowJo^TM^ V10 software (BD Biosciences, Franklin Laker, NJ, USA). Up to fluorescence minus four controls were used to define the gating strategy. For CD73 and PD-L1 staining, isotype controls were used (Table S2).

### 4.5. Enzyme-Linked Immunosorbent Assay (ELISA)

10^6^ Ret or SVEC4-10 cells were cultured in T25 in DMEM (PAN-Biotech GmbH, Aidenbach, Germany) with 10% heat-inactivated FBS (Merck KGaA, Darmstadt, Germany) and 1% penicillin/streptomycin (Thermo Fisher Scientific, Waltham, MA, USA) for 24 h. After washing with PBS, cells were cultured for an additional 24 h in DMEM medium without FBS. Medium was collected and sterile filtered (0.22 µm). The Mouse CXCL1/KC DuoSet (Research And Diagnostic Systems, Inc., Minneapolis, MN, USA) was used according to the manufacturer’s instructions to determine the concentration of CXCL1.

### 4.6. Bio-Plex Assay

Mouse plasma was prepared through centrifugation of EDTA-treated peripheral blood at 1000× *g* for 10 min at 4 °C. Supernatant was collected and filtered using a 0.22 µm filter and stored at −20 °C. Tumor samples were snap frozen in liquid nitrogen and stored at −80 °C. For the lysis of tumor samples, they were treated with cell lysis kit (Bio-Rad Laboratories, Inc., Hercules, CA, USA) according to the manufacturer’s instructions. Protein amount in lysates and plasma was measured by Pierce BCA protein assay kit (Thermo Fisher Scientific, Waltham, MA, USA). Protein concentration in lysates was adjusted to 1 mg/mL with serum diluent (Bio-Rad Laboratories, Inc., Hercules, CA, USA). Chemokine levels in plasma and tumor tissue were analyzed by multiplex technology (Bio-Rad Laboratories, Inc., Hercules, CA, USA) the Pro Mouse Chemokine 31-plex kit according to the manufacturer’s instruction. Acquisition and data analysis were performed by bio-plex Manager™.

### 4.7. Inhibition of T Cell Proliferation Assay

CD8^+^ splenic T cells isolated from healthy C57BL/6 mice using magnetic-activated cell sorting (MACS^®^, Miltenyi Biotec B.V. & Co. KG, Bergisch Gladbach, Germany, Cat. 130-104-075, cell purity was more than 95%) were stained with 2 nM carboxyfluorescein succinimidyl ester (CFSE) (BioLegend, San Diego, CA, USA) in PBS (10^6^ cells/mL) followed by the incubation for 5 min at 37 °C. T cells were washed with PBS at 300 g for 5 min and resuspended in RPMI-1640 with GlutaMAX supplemented with 10% heat-inactivated FBS, 1% penicillin/streptomycin, 10 mM HEPES, 1 mM sodium pyruvate, 50 µM β-mercaptoethanol, 1 mM MEM non-essential amino acids (all Thermo Fisher Scientific, Waltham, MA, USA). T cells were cultured alone or with CD11b^+^Gr1^+^ IMC, M-MDSC or PMN-MDSC at a T cell to IMC/MDSC ratio of 1:2 in a 96 well round bottom plate. To induce T cell proliferation, the plate was coated with 100 µL of an anti-CD28 and anti-CD3 mAb (both eBioscience, Inc., San Diego, CA, USA) in PBS for 3 h at 37 °C and 5% CO_2_. Unstimulated T cells served as a negative control. Samples were stained for CD8^+^ T cells after 96 h of culture and CFSE expression in these cells was determined by flow cytometry.

### 4.8. In Vitro Migration of MDSC Subsets from RET Transgenic Mice

5 × 10^4^–2.5 × 10^5^ M-MDSC or PMN-MDSC were seeded in 200 µL DMEM medium in the upper chamber of a polycarbonate Transwell culture insert (Thermo Fisher Scientific, Waltham, MA, USA). The lower chamber was filled with 500 µL medium with or without CXCL1 (100 ng/mL) or CXCL5 (100 ng/mL) (both from PeproTech, Inc., Rocky Hill, NJ, USA). The percentage of migrated cells from the upper to the lower compartment was determined after 24 h of incubation at 37 °C and 5% CO_2_ by flow cytometry. This migration assay was also performed with lymphatic endothelial cells present on the transwell insert. Transwell inserts were coated with 100 µL of 0.2% gelatin in PBS. Then, 24 h after, 1 × 10^5^ lymphatic endothelial SVEC4-10 cells were seeded in 200 µL in the upper compartment of the inserts. The lower chamber was filled with 500 µL medium. Upon 24 h of incubation, medium in the upper compartment was removed and inserts were transferred to a new 24 well plate followed by addition of the cells and compounds to the lower and upper compartment. To test migration of PMN-MDSC towards Ret melanoma cells over an endothelial cell layer, 1 × 10^5^ cells were seeded in 500 µL medium in a 24 well plate. After 24 h of incubation, inserts with grown endothelial cell layer were placed in wells containing Ret cells followed by the measurement of PMN-MDSC migration towards Ret melanoma cells during another 24 h cells. Cell migration was tested in the presence of the anti-CXCR2 inhibitor SB265610 (100 nM) in the upper and lower compartment.

### 4.9. Co-Culture of MDSC Subsets with NK Cells

MDSC were generated as previously described [56]. Briefly, 2.5 × 10^6^ C57BL/6 BM cells were cultured in RPMI-1640 with GlutaMAX supplemented with 10% heat-inactivated FBS, 1% penicillin/streptomycin, 10 mM HEPES, 1 mM sodium pyruvate, 50 µM β-mercaptoethanol, 1 mM MEM non-essential amino acids (all Thermo Fisher Scientific, Waltham, MA, USA), 40 ng/mL GM-CSF, and 40 ng/mL IL-6 (PeproTech, Inc., Rocky Hill, NJ, USA) for 4 days. Then PMN-MDSC and M-MDSC were isolated from the culture using magnetic-activated cell sorting (MACS^®^, Miltenyi Biotec B.V. & Co. KG, Bergisch Gladbach, Germany, Cat. 130-094-538, cell purity was more than 90%) according to the manufacturer’s instructions. NK cells were isolated from the spleens of normal C57BL/6 mice using MACS^®^ (MACS^®^, Miltenyi Biotec B.V. & Co. KG, Bergisch Gladbach, Germany, Cat. 130-115-818, the purity of isolated cell was more than 90%) according to the manufacturer’s instructions and stimulated with IL-2 (0.1 ng/mL), IL-12 (1 ng/mL), IL-18 p80 (20 ng/mL) (all from PeproTech, Inc., Rocky Hill, NJ, USA) for 3 h. Afterwards, NK cells were washed and cultured alone or with PMN-MDSC or M-MDSC for 4 h followed by the staining of NK cells for intracellular IL-16 and IFN-γ and measurement by flow cytometry.

### 4.10. Transplantation of AB12 Mesothelioma Cells

Five-six weeks old female Balb/c mice were injected s.c. into the flank with 1.5 × 10^6^ AB12 cells. Established tumors were removed, weighted and treated in L15 medium supplemented with 0.17 mg/mL collagenase I, 0.05 mg/mL collagenase II, 0.17 mg/mL collagenase IV, 0.02 mg/mL elastase (all from Worthington Biochemical Corp., Lakewood, NJ, USA) and 0.02 mg/mL DNase (Merck KGaA, Darmstadt, Germany) at 37 °C for 30 min. Red blood cell (RBC) lysis was performed with RBC lysis buffer (Biological Industries, Beit HaEmek, Israel).

### 4.11. Therapy of Melanoma Bearing Mice

Upon detection of first macroscopic tumor lesions, *RET* transgenic mice received either the CXCR2 inhibitor SB265610 (2 mg/kg, Research And Diagnostic Systems, Inc., Minneapolis, MN, USA) or DMSO control solution (5% in PBS) i.p., twice a week for four weeks. Mice were monitored two times per week for tumor progression for 100 days after therapy initiation. For analysis of tumor infiltrating immune cells, mice were sacrificed one day after the last injection.

### 4.12. Therapy of Mesothelioma-Bearing Mice

Ten days following s.c. injection of AB12 cells, tumor bearing mice were treated with the CXCR2 inhibitor SB265610 (2 mg/kg, Cayman Chemical, Ann Arbor, MI, USA) or with DMSO control solution i.p. every second days. Mice were sacrificed nine days after the first injection. Tumors were harvested, weighted, and processed for further analysis as described above.

### 4.13. Transplantation of Primary Skin Melanomas and Adjuvant Therapy

Primary melanoma fragments from *RET* transgenic mice were implanted as previously described [57]. Briefly, tumor fragments were implanted intradermally (i.d.) into C57BL/6 mice. Upon engraftment, the fragments were cryopreserved. Then they were implanted i.d. at the ventral side of C57BL/6 mice. At day 14–21 upon implantation, the tumors were resected (after they reached a diameter of approximately 1 cm). Mice were monitored 2–3 times per week. Seven days after resection of the primary tumor, mice were injected i.p. with the anti-CXCR2 inhibitor SB265610 or with DMSO control solution twice a week for four to six weeks. To investigate pre-metastatic niches, one day after the last injection normal, non-metastatic lung, LN and liver tissue as well as visible macrometastases were isolated followed by the analysis of prepared single cell suspensions by flow cytometry.

### 4.14. TCGA Data

Data on survival of skin cutaneous melanoma patients and expression of CXCL1 and CXCL5 was taken from the TCGA database and assessed using OncoLnc [58]. Melanoma patients (*n* = 458) were stratified into those with low and high expression of CXCL1 and CXCL5 (lower and upper percentile 50). Statistical significance for the overall survival was determined using a Cox regression analysis.

### 4.15. Statistical Analysis

Statistical analysis of data was performed using the GraphPad Prism software (GraphPad Software, San Diego, CA, USA) in at least three biological replicates or at least three independent experiments. Two groups were compared with the paired or unpaired two-tailed Student’s t test assuming a Gaussian distribution of the data. Correlation analysis was done using linear regression analysis. Survival curves were generated using the Kaplan–Meier method and statistical comparison was done by the Logrank (Mantel–Cox) test. A value of *p* < 0.05 was considered statistically significant.

## 5. Conclusions

Taken together, our findings imply PMN-MDSC as one of the major drivers of primary melanoma growth and metastatic formation. PMN-MDSC were demonstrated to infiltrate primary tumors and metastatic lesions via CXCR2/CXCL1 interactions. The anti-CXCR2 therapy was able to delay the development of primary tumors and metastases in LN and distant organs by reducing the accumulation of PMN-MDSC in melanoma microenvironment. The observed therapeutic effect in melanoma bearing mice was associated with the accumulation of NK cells while no involvement of effector T cells in response to anti-CXCR2 therapy was found. We suggest that the blocking of PMN-MDSC recruitment improve the efficiency of immunotherapy of melanoma patients.

## Data Availability

The data presented in this study are available on request from the corresponding author.

## Supplementary Materials

The following are available online at https://www.mdpi.com/2072-6694/13/4/726/s1, Figure S1: Gating strategy for MDSC in primary melanomas, Figure S2: Tumor infiltrating M-MDSC and tumor progression, Figure S3: Representative dot plots for the expression of immunosuppressive markers on MDSC subsets, Figure S4: Chemokine receptor expression on mesothelioma infiltrating MDSC subsets, Figure S5: Role of CXCL5 in survival of melanoma patients and migration of PMN-MDSC in melanoma-bearing mice, Figure S6: Role of CXCR2/CXCL1 and CXCR2/CXCL5 interaction in M-MDSC migration, Figure S7: Effect of the CXCR2 inhibition on mesothelioma development, Figure S8: Effect of the anti-CXCR2 therapy on endothelial cells in skin tumors from RET transgenic mice one day after the last injection, Figure S9: Effect of CXCR2 inhibitor therapy on PMN-MDSC frequency in RET transgenic mice, Table S1: Fluorescence conjugated monoclonal antibodies for flow cytometry, Table S2: Isotype controls for flow cytometry.
