# Supplementary material for: Blocking Migration of Polymorphonuclear Myeloid-Derived Suppressor Cells Inhibits Mouse Melanoma Progression
Source: Cancers (Basel). 2021 Feb 10;13(4):726. doi: 10.3390/cancers13040726 (PMC7916588; doi:10.3390/cancers13040726)
Supplement: Supplementary file 1 [file cancers-13-00726-s001.pdf]

Supplementary Materials: Blocking Migration of Polymorphonuclear Myeloid-Derived Suppressor Cells Inhibits Mouse Melanoma Progression

Christopher Groth, Ludovica Arpinati, Merav E Shaul, Nina Winkler, Klara Diester, Nicolas Gengenbacher, Rebekka Weber, Ihor Arkhypov, Samantha Lasser, Vera Petrova, Hellmut G. Augustin, Peter Altevogt, Jochen Utikal, Zvi G. Fridlender and Viktor Umansky

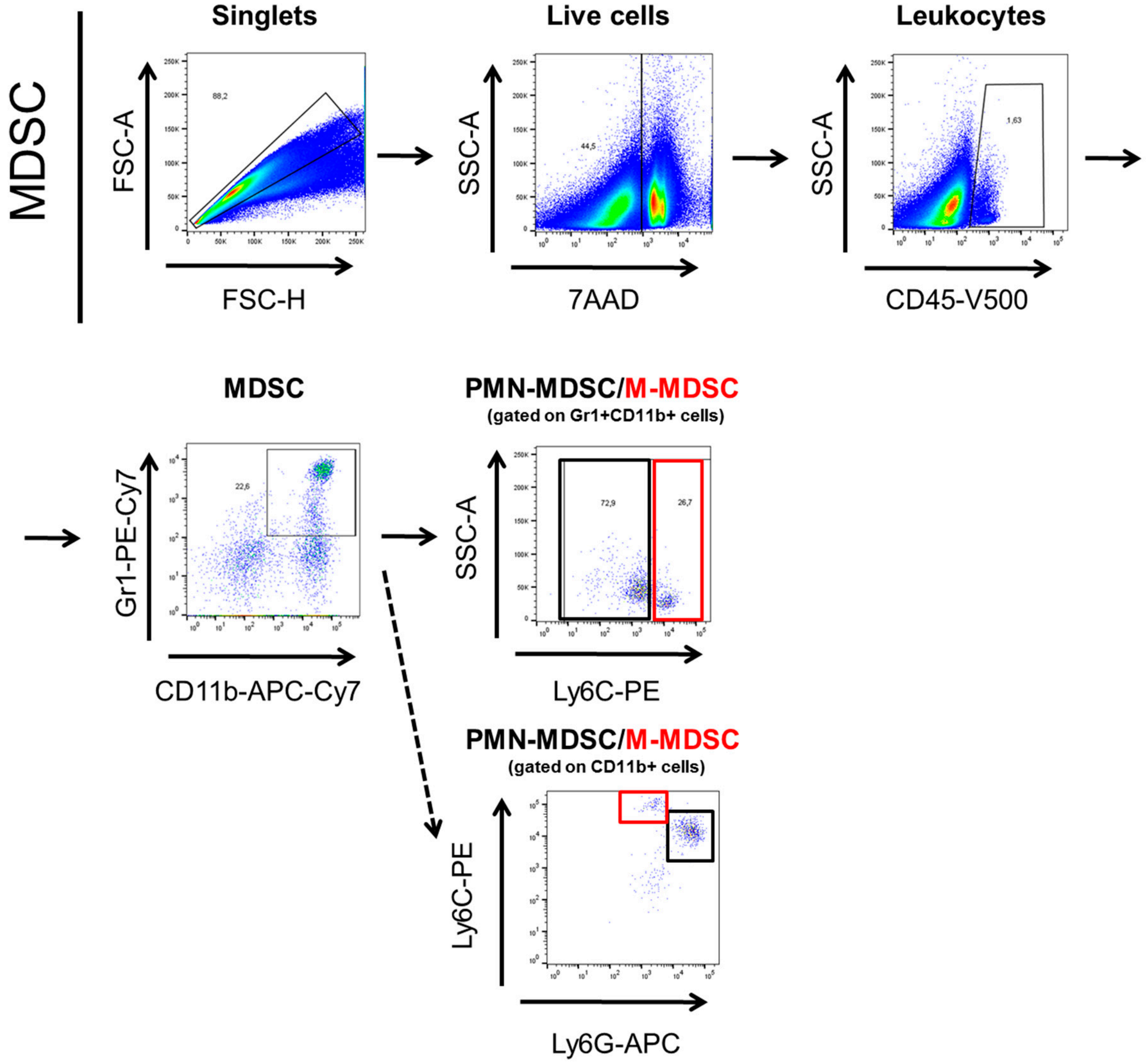

Figure S1. Gating strategy for MDSC in primary melanomas. After duplet exclusion, PMN-MDSC and M-MDSC were gated as $7AAD^{-}CD45^{+}CD11b^{+}Ly6G^{+}Ly6C^{lo}$ or $7AAD^{-}CD45^{+}CD11b^{+}Ly6G^{-}Ly6C^{hi}$ cells respectively. Alternatively, PMN-MDSC and M-MDSC were gated as $7AAD^{-}CD45^{+}CD11b^{+}Gr1^{+}Ly6C^{lo}$ or $7AAD^{-}CD45^{+}CD11b^{+}Gr1^{+}Ly6C^{hi}$ cells respectively.

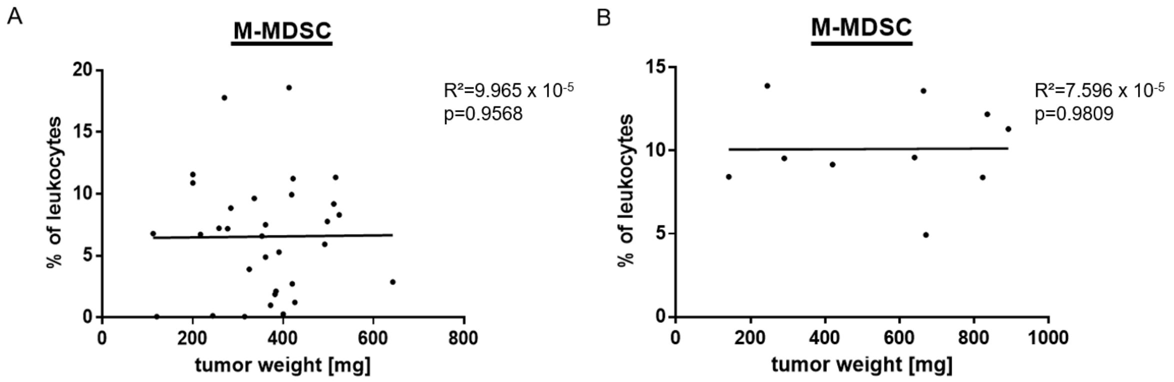

Figure S2. Tumor infiltrating M-MDSC and tumor progression. The weight of each melanoma (**A**) or mesothelioma (**B**) sample in mg was plotted against the percentage of M-MDSC among $CD45^+$ leukocytes in tumors from respective mice measured by flow cytometry (n = 10–32). The correlation was evaluated by a linear regression analysis.

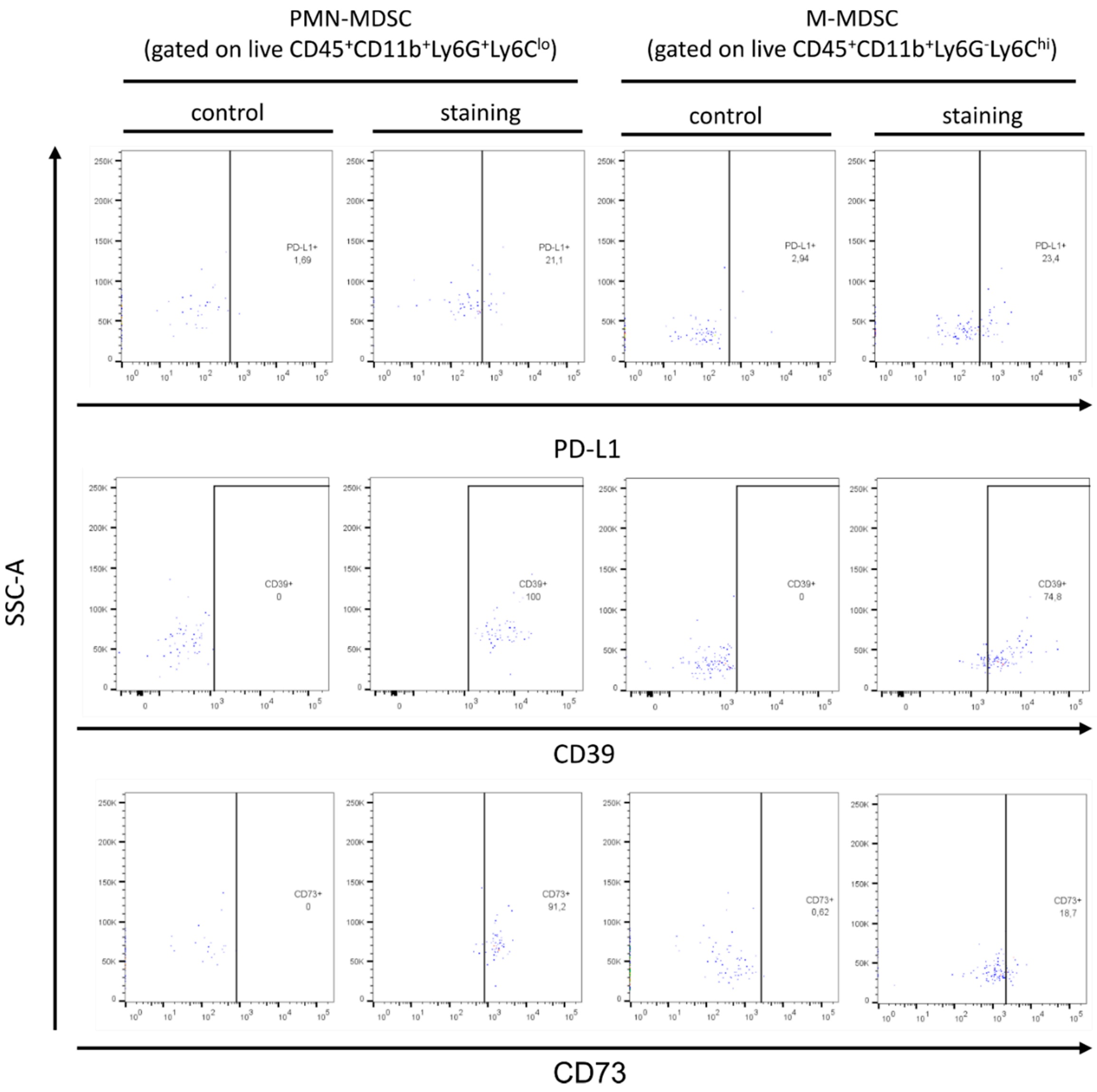

Figure S3. Representative dot plots for the expression of immunosuppressive markers on MDSC subsets. Representative control stainings and stainings for the expression of PD-L1, CD39 and CD73 on PMN-MDSC and M-MDSC infiltrating RET melanomas.

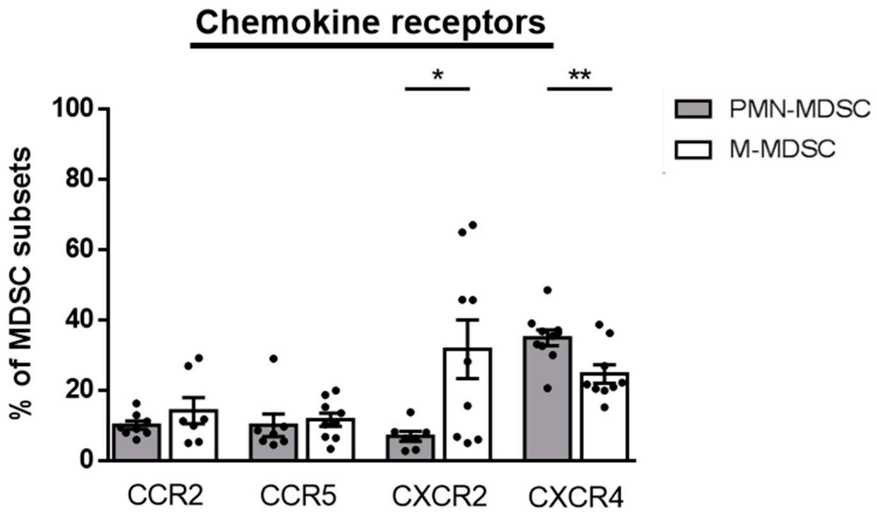

Figure S4. Chemokine receptor expression on mesothelioma infiltrating MDSC subsets. Expression of CCR2, CCR5, CXCR2 and CXCR4 on PMN- and M-MDSC from mesothelioma-bearing mice was detected by flow cytometry. Data are shown as the percentage cells positive for chemokine receptors among respective MDSC subsets (mean ± SEM; n = 7–10 mice/group). * $p < 0.05$, ** $p < 0.01$.

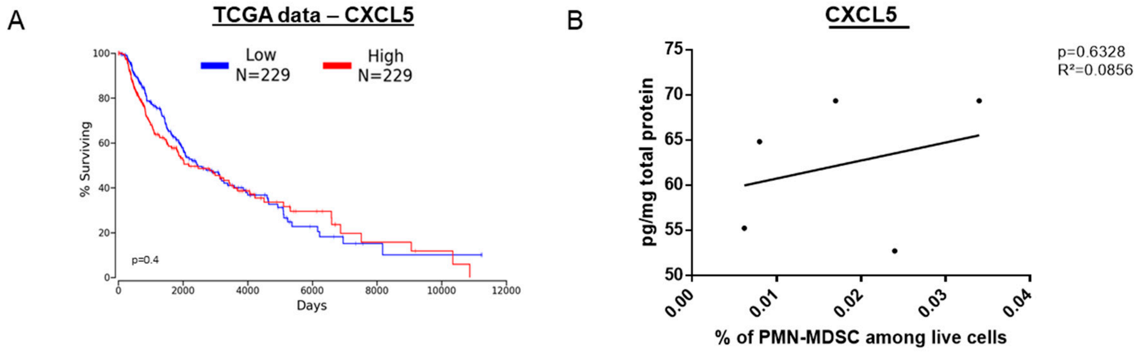

Figure S5. Role of CXCL5 in survival of melanoma patients and migration of PMN-MDSC in melanoma bearing mice. (A) Overall survival analysis of melanoma patients stratified in groups with low and high CXCL5 expression (lower and upper percentile 50). (B) The intratumoral levels of CXCL5 expressed as pg/mg protein were plotted against the percentage of tumor-infiltrating PMN-MDSC among total live cells from respective mice (n = 5). The correlation was evaluated by a linear regression analysis.

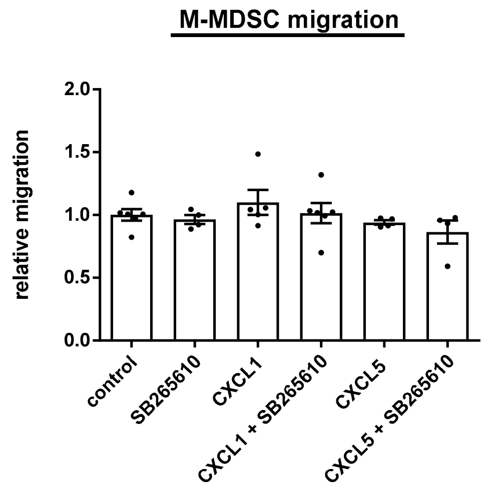

Figure S6. Effect of CXCL1 and CXCL5 on the migration of M-MDSC. M-MDSC were isolated from the BM of tumor bearing RET transgenic mice. Spontaneous migration (control) and migration towards CXCL1 (100 ng/mL) or CXCL5 (100 ng/mL) was determined in the presence and absence of the CXCR2 inhibitor SB265610 (100 nM) after the incubation for 24 h in a transwell assay. Data are normalized to the spontaneous migration of M-MDSC (control) (mean ± SEM; *n* = 4–6).

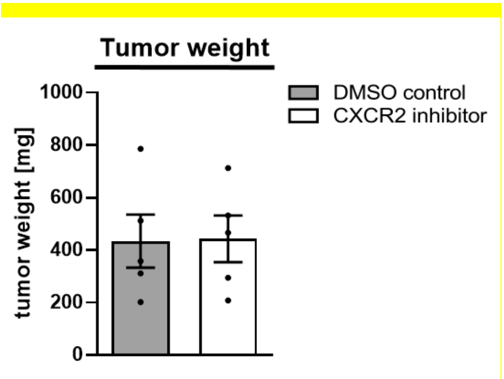

Figure S7. Effect of the CXCR2 inhibition on mesothelioma development. Balb/c mice were injected i.p. with the CXCR2 inhibitor SB265610 (2 mg/kg) or the control DMSO solution (control group) i.p. every second days starting on day 10 after tumor cell injection. Tumors were removed nine days after the first injection. Data are presented as tumor weight in mg (mean ± SEM; *n* = 5 mice/group).

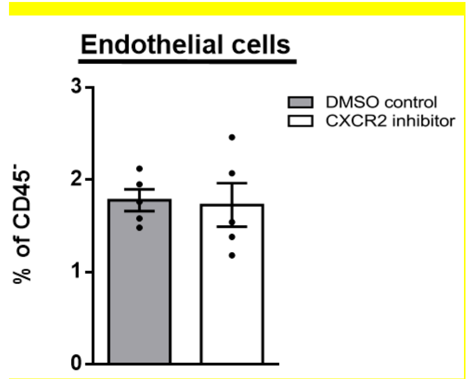

Figure S8. Effect of the anti-CXCR2 therapy on endothelial cells in skin tumors from RET transgenic mice one day after the last injection. Frequency of $CD31^+$ cells was measured by flow cytometry. Data are shown as the percentage of $CD31^+$ endothelial cells among $CD45^-$ cells (mean ± SEM; *n* = 5 mice/group).

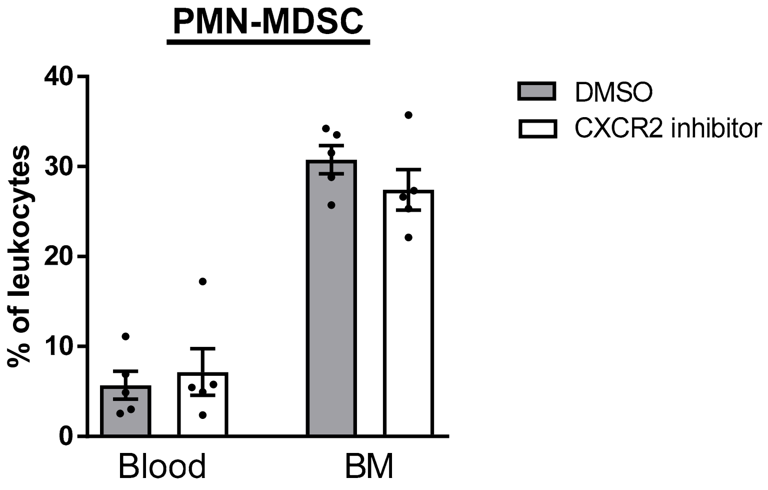

Figure S9. Effect of CXCR2 inhibitor therapy on PMN-MDSC frequency in RET transgenic mice. RET transgenic melanoma bearing mice were injected i.p. with the CXCR2 inhibitor SB265610 (2 mg/kg) or the control DMSO solution (control group) twice a week for four weeks. One day after the last injection, PMN-MDSC frequencies within $CD45^+$ leukocytes in peripheral blood and bone marrow (BM) (mean ± SEM; 5 mice/group) were determined by flow cytometry.

Table S1. Fluorescence conjugated monoclonal antibodies for flow cytometry.

Specificity	Conjugate	Clone	Manufacturer	Order no.	Dilution
CCR2	BV605	475301	BD Bioscience	747969	1:100
CCR2	APC	REA538	Miltenyi Biotec	130119658	1:50
CCR4	PE-Cy7	2G12	Biolegend	131213	1:100
CCR5	AF488	HM-CCR5	Biolegend	107008	1:100
CCR5	APC	REA354	Miltenyi Biotec	130125222	1:50
CCR5	BB515	C34-3448	BD Bioscience	565093	1:100
CD11b	APC-Cy7	M1/70	BD Bioscience	557657	1:200
CD11c	APC-Cy7	HL3	BD Bioscience	561119	1:100
CD3	PerCP-Cy5.5	145-2C11	BD Bioscience	551163	1:100
CD3	APC	145-2C11	BD Bioscience	553066	1:50
CD4	PE	RM4-5	BD Bioscience	552775	1:100
CD4e	APC	GK1.5	Miltenyi Biotec	130116523	1:50
CD8a	PE-Cy7	RPA-T8	BD Bioscience	557746	1:100
CD8a	eF450	53-6.7	Invitrogen	48008182	1:200
CD25	APC-Cy7	PC61	BD Bioscience	564458	1:100
CD31	PE	390	Biolegend	102408	1:100
CD39	PE-Cy7	Duha59	Biolegend	143801	1:100
CD45	V500	30-F11	BD Bioscience	561487	1:100
CD45	FITC	30-F11	Biogems	0751250100	1:100
CD69	APC	H1.2F3	BD Bioscience	561932	1:100
CD73	BV605	TY/11.8	Biolegend	127215	1:100
CXCR2	FITC	SA044G4	Biolegend	149309	1:100
CXCR2	APC	REA538	Miltenyi Biotech	130119658	1:50
CXCR4	BV421	2B11	BD Bioscience	562738	1:100
CXCR4	PE	2B11	Biogems	169126050	1:25
F4/80	APC	T45-2342	BD Bioscience	566787	1:100
FoxP3	FITC	FJK-16s	Invitrogen	11577380	1:100
Gr1	PE-Cy7	RB6-8C5	BD Bioscience	565033	1:600
IL-16	PE	14.1	Biolegend	519106	1:100

IFN-γ	FITC	XMG1.2	Biolegend	505805	1:100
Ly6C	PE	AL-21	BD Bioscience	560592	1:100
Ly6C	VioBlue	REA796	Miltenyi Biotech	130111921	1:50
Ly6G	APC	1A8	BD Bioscience	560599	1:100
Ly6G	Violet450	1A8	Biogems	831140100	1:100
NK1.1	BV605	PK136	Biolegend	108739	1:100
NKp46	PE	29A1.4	Biogems	374126025	1:50
PD-1	BV421	EH12.1	BD Bioscience	562584	1:100
PD-L1	BV421	MIH5	BD Bioscience	564716	1:100

Table S2. Isotype controls for flow cytometry.

Isotype	Conjugate	Clone	Manufacturer	Order No.	Dilution
Hamster IgG	AF488	HTK888	Biolegend	400923	1:100
Rat IgG1, κ	BV605	R3-34	BD Bioscience	562993	1:100
Rat IgG2a	BV421	B39-4	BD Bioscience	562965	1:100
